# A risk predictor of restenosis after superficial femoral artery stent implantation: relevance of mean platelet volume

**DOI:** 10.1186/s12872-020-01633-8

**Published:** 2020-08-08

**Authors:** Yao Bo Yang, Jing Shen, Sheng Hai Wang, Jian Bo Song, Fangfang Ge, Jia Pei Xie, Jiang Shuai Qu, Xin Zu Mao, Zhao Cheng Kuang, Nan Shang, Xiang Wang, Ye Jun Wu, Fan Yang, Yue Yuan, Hongxin Wang, Jun Sun, Jicheng Fang, Liang Xiao

**Affiliations:** 1 Department of Intervention, The Fourth Affiliated Hospital of China Medical University, 4 Chongshan Road, Huanggu District, Shenyang, 110000 Liaoning China; 2 Shanxi provincial people’s Hospital, Taiyuan, Shanxi China; 3 Yan’an People’s Hospital, Yan’an, Shaanxi China; 4 The First Affiliated Hospital of Dalian Medical University, Dalian, Liaoning China

**Keywords:** Risk predictor, Superficial femoral artery, Mean platelet volume, In-stent restenosis

## Abstract

**Background:**

To investigate the relationship between an increase in the pre- and post-operative mean platelet volume (MPV) and superficial femoral artery in-stent restenosis (ISR) rate.

**Methods and results:**

We recruited patients that underwent superficial femoral artery stenting for lower extremity arteriosclerosis obliterans at our hospital from March 2015 to March 2018. All patients gave venous blood three days before and following implantation. Doppler ultrasound, computed tomography angiography or digital subtraction angiography were used for regular follow-up examination. Logistic regression was used to identify predictors of ISR after superficial femoral artery stenting. We enrolled 173 patients, of which 34 (19.6%) were determined as having ISR for a mean of 8.9 ± 2.7 months (3–12 months). Neutrophil count, neutrophil ratio, lymphocyte ratio and platelet count pre-implantation, and platelet count and MPV after stent implantation, and the pre- and post-operative mean platelet volume difference (MPVD) and mean platelet volume difference ratio (MPVDR) were all statistically different when comparing the ISR and non-restenosis groups (*p* < 0.05). A positive correlation was found for post-operative MPV and presence of ISR (r = 0.58; *P* < 0.001). A MPVD not less than 1.5 fL was associated with an odds ratio of 9.17 (95% CI [3.76 to 22.35]; *P* < 0.001) for presence of ISR. A MPVDR of not less than 17.9% was associated with an odds ratio of 7.68 (95% CI [3.19 to 18.49]; *P* < 0.001) for occurrence of ISR.

**Conclusions:**

An increase in pre- and post-operative MPV was correlated with the occurrence of superficial femoral artery ISR.

## Background

Arteriosclerosis obliterans of the lower limbs is caused by the formation of atherosclerotic plaques of the lower limbs, which leads to arterial stenosis and occlusion, leading to chronic limb ischemia. With the aggregation of an aging population, the incidence of peripheral vascular disease is increasing, especially in the elderly, where the incidence can reach 15–20% [1]. Percutaneous transluminal stent implantation is an accepted technique for the treatment of superficial femoral artery occlusive disease [2–4]. The use of percutaneous transluminal stent implantation to revascularize the superficial femoral artery can result in an initial technical success rate of more than 95% [5]. The greater use of percutaneous transluminal stent implantation within the superficial femoral artery (superficial femoral artery stenting or SFAS) is primarily due to a decreased incidence in operative morbidity and mortality, decreased length of hospital stay, and decreased wound complications. However, one-year ISR occurs in approximately 18–40% of patients that elect to undergo SFAS [6]. However, this risk is detected in the clinical setting in a timely manner. Therefore, it is particularly important to determine the risk factors of restenosis in superficial femoral artery stents early in clinical treatment by simple and convenient examination methods.

Platelets, by their capacity to adhere to the sites of an arterial injury, can form aggregates and secrete platelet-derived growth factors (PDGF), and appear to play an important role in neointimal hyperplasia and the appearance of restenosis [7–10]. The platelet volume is a marker of platelet function and activation [11]. Several experimental studies have indicated that large platelets contain more α-granules [12], produce more prothrombotic factors like thromboxaneB2 [13, 14], and release more serotonin and h-thromboglobulin [12, 15] than smaller platelets. A large MPV is a predictive marker of in-stent restenosis after coronary [16, 17] and carotid stenting [18]. However, the utility of MPV as a predictor of risk for superficial femoral artery restenosis has not been previously reported in the literature. The aim of this study was to determine the relationship between the pre- and post-operative difference in MPV and the superficial femoral artery ISR rate.

## Methods

We recruited 173 patients that underwent SFAS for lower extremity arteriosclerosis obliterans at our hospital from March 2015 to March 2018. All patients donated venous blood three days before and then after implantation. Routine hematological parameters including erythrocytic count, hemoglobin, leukocyte count, neutrophil count, neutrophil ratio, lymphocyte count, lymphocyte ratio, eosinophil count, eosinophil ratio, basophil count, basophils ratio, platelet distribution width, platelet count, mean platelet volume, uric acid levels, creatinine levels, and levels of triglycerides, cholesterol, high-density lipoprotein, low-density lipoprotein, and serum glucose were measured by an auto-analyzer (Model XE2100; Sysmex Co, Kobe, Japan). Clinical and demographic data, and laboratory results were obtained from the hospital electronic medical records system for admitted patients. Hypertension was defined as blood pressure ≥ 140/90 mm/Hg or treatment with anti-hypertensive medications. Diabetes mellitus was defined as fasting glucose ≥126 mg/dL or treatment with oral anti-diabetic drugs or insulin. Smokers were defined as current cigarette users or patients who had quit smoking within 1 month of the procedure. Inclusion criteria included the following: 1. adult patients that underwent successful percutaneous transluminal stent implantation for superficial femoral artery lesions; 2. TASC-II classification of the femoral artery [19]: TASC-IIA, TASC-IIB, and TASC-IIC patients; 3. at least one arterial run-off below the knee, although stenosis lesions that were not limiting the flow may be included; 4. No evidence of residual inflow problems in the aorto-iliac artery, although stenosis lesions that were not limiting the flow may be included. Exclusion criteria included the following: 1. evidence of contraindications to anti-coagulation; 2. evidence of hematological disease; 3. presence of severe cardiac insufficiency (New York Heart Association grade III or IV), liver dysfunction (Child grade B or C) or renal insufficiency (creatinine clearance < 30 mL/min); and 4. no arterial run-off below the knee. All patients received 5000 units of heparin during the procedures. After taking 100 mg of aspirin and clopidogrel at 75 mg for 12 months, clopidogrel was discontinued and administration of aspirin was sustained. Patients with hypertension, diabetes, coronary heart disease and hyperlipidemia were given symptomatic treatment such as antihypertensive, hypoglycemic, cardiac and lipid lowering. Doppler ultrasonography, computed tomography angiography or digital subtraction angiography were performed every 3–6 months after stenting the superficial femoral artery. ISR was defined as not less than 50% stenosis in the treated lesion [20]. The study was approved by the local Ethics Committee of the Fourth Affiliated Hospital of China Medical University. All patients signed an informed consent.

### Statistical analysis

Data were analyzed using the SPSS version 21.0 software package. Continuous data were given as mean ± S.D. Discrete parameters were presented as percentages. Categorical variables were tested using the Chi-square test and Fisher’s exact test. Receiver operating characteristic (ROC) curve analysis was used to determine the cutoff values of the MPVD and MPVDR. Linear and Spearman’s rank regressions were used with the ISR as the dependent variable. Logistic regression analysis was used to identify predictors of ISR. A linear relationship between an increase in the MPV and the onset time of restenosis was assessed by calculating the Pearson’s correlation coefficient. Kaplan–Meier analysis was performed to compare cumulative risk of ISR between groups. A probability (*P*) value of < 0.05 was considered statistically significant.

## Results

We enrolled 173 patients to this study, with a mean age of 69.5 ± 11.72 (45–90 years). There were 135 (78%) men and 38 (22%) women in this group. ISR was detected in 34 (19.6%) patients after a mean of 8.9 ± 2.7 months (range 3–12 months). There was no difference between groups for age and sex distribution. There were 95 (54.91%) diabetes mellitus, 93 (53.76%) smoking, 47 (27.12%) coronary heart disease, and 97 (56.07%) hypertension patients although none were statistically different when comparing the ISR and no-restenosis groups (*P* > 0.05). In addition, the TASC II classification of the femoral artery was not statistically different between the ISR and no-restenosis groups (*P* > 0.05; Table 1).

**Table 1:** Baseline characteristics of the 173 patients

	Total, n = 173 (%)	Restenosis, *n* = 34 (%)	No restenosis, *n* = 139 (%)	*P* value
Male	135 (78.0)	27 (79.4)	108 (77.7)	0.86
Age, Y	69.54 ± 10.50	67.53 ± 11.62	70.03 ± 10.19	0.21
Smoking	93 (53.76)	18 (52.94)	75 (53.96)	0.39
Hypertension	97 (56.07)	18 (52.94)	79 (56.83)	0.66
Diabetes mellitus	95 (54.91)	16 (47.06)	79 (56.83)	0.10
Statin treatment	38 (21.97)	8 (23.53)	30 (21.58)	0.81
CAD	47 (27.12)	10 (29.41)	37 (34.26)	0.74
TASC II classification				
TASC A	9	3	6	0.33
TASC B	88	14	74	
TASC C	76	17	59	

Key: *CAD* coronary artery disease

Univariate analysis showed that the pre-operative serum glucose, uric acid, creatinine, total cholesterol, triglyceride, and HDL and LDL levels, were not statistically different between the ISR and no-restenosis groups (*P* > 0.05). The post-operative creatinine and uric acid levels were not statistically different between the ISR and no-restenosis groups (*P* > 0.05; Table 2).

**Table 2:** Comparison of biochemical parameters between two groups before procedures

	Total, n = 173	Restenosis, n = 34	No restenosis, n = 139	*P* value
Serum glucose, mmol/L	7.35 ± 2.80	6.79 ± 2.30	7.50 ± 2.90	0.18
Uric acid, umol/L	333.18 ± 116.6	365.17 ± 112.7	327.56 ± 117.24	0.2
Creatinine, umol/L	84.58 ± 30.61	86.00 ± 30.17	84.24 ± 30.82	0.76
Total cholesterol, mmol/L	4.29 ± 1.25	4.33 ± 1.47	4.27 ± 1.19	0.79
Triglyceride, ummol/L	1.87 ± 1.71	2.09 ± 1.89	1.82 ± 1.67	0.41
HDL, mmol/L	1.12 ± 1.33	1.45 ± 2.93	1.04 ± 0.32	0.11
LDL, mmol/L	2.69 ± 1.00	2.62 ± 1.17	2.7 ± 0.96	0.64
Uric acid, umol/L	271.76 ± 99.09	286.63 ± 84.42	268.12 ± 102.3	0.33
Creatinine, umol/L	91.49 ± 25.7	88.94 ± 21.51	91.87 ± 26.68	0.69

Key: *HDL* high-density lipoprotein, *LDL* low-density lipoprotein

The preoperative neutrophil count (*P* = 0.038), the pre-operative neutrophil ratio (*P* = 0.01), the pre-operative lymphocyte ratio (*P* = 0.03), and the pre-operative platelet count (*P* = 0.04) were all statistically different between the ISR and no-restenosis groups (Table 3). The post-operative platelet count (*P* < 0.001) and the post-operative MPV (*P* < 0.001) were statistically different between both groups. In addition, the pre- and post-operative platelet count difference (*P* = 0.01), the pre- and post-operative mean platelet volume difference (*P* < 0.001), and the pre- and post-operative mean platelet volume difference ratio (*P* < 0.001) were all statistically different between the ISR and no-restenosis groups (Table 4).

**Table 3:** Comparison of blood parameters between two groups preoperative

	Total, *n* = 173	Restenosis, N = 34	No restenosis, *n* = 139	*P* value
Erythrocytic,× 10^12^/L	4.52 ± 0.57	4.47 ± 0.53	4.53 ± 0.59	0.57
Hemoglobin, g/L	141.4 ± 13.6	138.23 ± 15.07	142.25 ± 13.19	0.12
Leukocyte,× 10^9^/L	8.31 ± 2.88	7.64 ± 1.66	8.48 ± 3.09	0.12
Neutrophil,× 10^9^/L	5.71 ± 2.81	4.82 ± 1.35	5.93 ± 3.03	0.04
Neutrophil ratio,%	66.88 ± 10.5	62.84 ± 8.81	67.87 ± 10.79	0.01
Lymphocyte,× 10^9^/L	1.88 ± 0.70	2.01 ± 0.66	1.85 ± 0.71	0.24
Lymphocyte ratio,%	24.10 ± 8.53	26.86 ± 8.26	23.42 ± 8.49	0.03
NLR	3.84 ± 0.85	2.84 ± 0.36	4.09 ± 0.43	0.02
Eosinophil,× 10^9^/L	0.18 ± 0.18	0.18 ± 0.14	0.18 ± 0.1	0.84
Eosinophil ratio,%	2.39 ± 2.13	2.36 ± 1.63	2.39 ± 2.34	0.93
Basophils,× 10^9^/L	0.03 ± 0.02	0.03 ± 0.01	0.03 ± 0.02	0.77
Basophils ratio,%	0.47 ± 0.23	0.51 ± 0.17	0.46 ± 0.25	0.31
PDW,%	16.76 ± 1.03	16.95 ± 0.66	16.71 ± 1.10	0.23
Platelet,× 10^9^/L	231.3 ± 86.7	204.31 ± 51.23	237.99 ± 92.37	0.04
MPV, fL	8.34 ± 0.83	8.49 ± 0.91	8.31 ± 0.82	0.24

Key: *PDW* platelet distribution width, *MPV* mean platelet volume, *NLR* neutrophil–lymphocyte ratio

**Table 4:** Comparison of blood parameters between two groups postoperative

	Total	Restenosis	No restenosis	*P* value
Erythrocytic,× 10^12^/L	4.51 ± 0.57	4.39 ± 0.52	4.54 ± 0.59	0.16
Hemoglobin, g/L	141.3 ± 13.4	141.17 ± 14.5	141.38 ± 13.24	0.93
Leukocyte,× 10^9^/L	8.43 ± 2.86	8.46 ± 2.23	8.42 ± 3.00	0.93
Neutrophil,× 10^9^/L	5.90 ± 2.69	5.83 ± 1.74	5.92 ± 2.88	0.80
Neutrophil ratio,%	68.73 ± 9.03	68.47 ± 8.37	68.80 ± 9.21	0.85
Lymphocyte,%	1.65 ± 0.60	1.71 ± 0.57	1.63 ± 0.61	0.52
Lymphocyte ratio,%	20.67 ± 6.88	20.77 ± 6.48	20.64 ± 7.00	0.92
NLR	9.88 ± 0.42	10.16 ± 0.88	9.81 ± 0.47	0.72
Eosinophil,× 10^9^/L	0.21 ± 0.17	0.21 ± 0.16	0.21 ± 0.18	0.98
Eosinophil ratio,%	2.48 ± 1.76	2.52 ± 1.86	2.47 ± 1.47	0.87
Basophils,× 10^9^/L	0.04 ± 0.05	0.03 ± 0.03	0.04 ± 0.05	0.24
Basophils ration,%	0.47 ± 0.42	0.40 ± 0.16	0.48 ± 0.47	0.30
PDW,%	17.41 ± 3.05	17.37 ± 1.04	17.42 ± 3.37	0.93
Platelet,× 10^9^/L	216.5 ± 81.3	168.2 ± 43.65	228.4 ± 84.06	0.001
MPV,fL	9.28 ± 0.78	10.04 ± 0.68	9.11 ± 0.79	< 0.001
PCD,× 10^9^/L	− 14.79 ± 54.16	−36.08 ± 46.23	−5.59 ± 54.82	0.01
MPVD, fL	0.90 ± 0.94	1.58 ± 1.07	0.75 ± 0.87	< 0.001
MPVDR,%	6.41 ± 12.19	19.24 ± 11.45	10.47 ± 11.92	< 0.001
MPVD ≥1.5 fL	48 (27.7)	22 (64.7)	26 (18.7)	< 0.001
MPVDR ≥17.9%	49 (28.3)	21 (61.8)	26 (20.1)	< 0.001

Key: *PDW* platelet distribution width, *MPV* mean platelet volume, *PCD* platelet count difference, *MPVD* mean platelet volume difference, *MPVDR* mean platelet volume difference ratio, *NLR* neutrophil–lymphocyte ratio

**Fig. 1:**
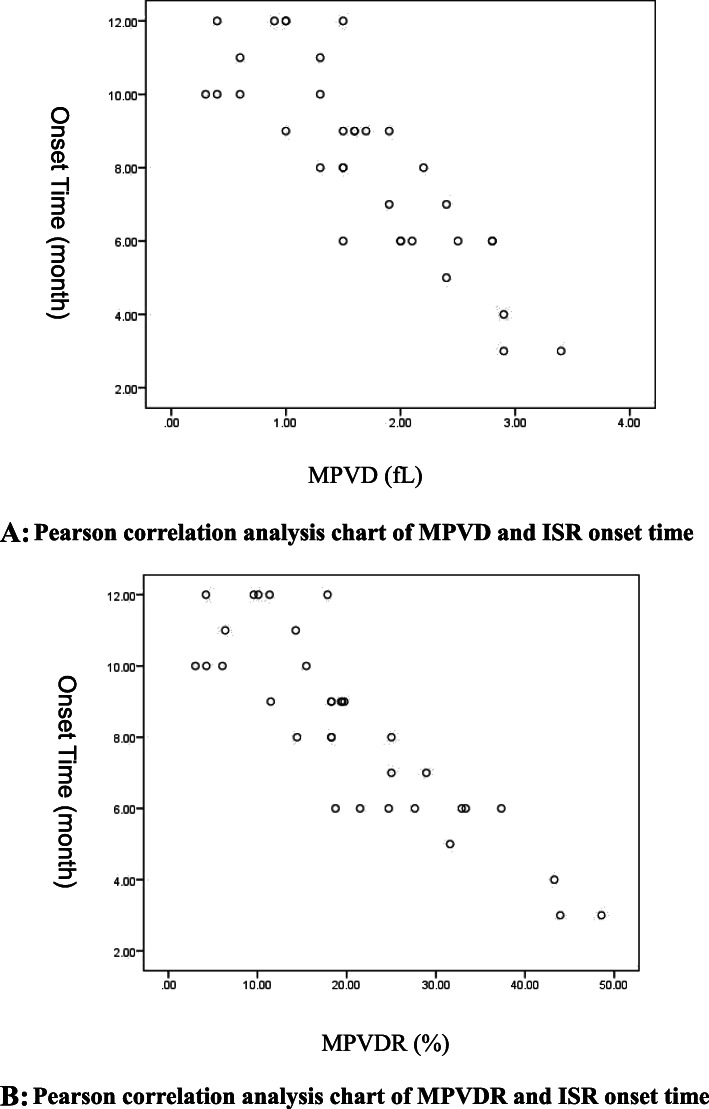
The ROC curve comparison of these two markers

A MPVD level not less than 1.5 fL predicted ISR with 64.7% sensitivity and 81.3% specificity for the prediction of ISR, as identified by the ROC curve. The area under the ROC curve (AUC) was 0.787 (95% CI [0.71–0.87], *p* < 0.001). A A MPVDR level not less than 17.9% predicted ISR with 64.7% sensitivity and 79.1% specificity, AUC was 0.764 (95% CI [0.68–0.85], p < 0.001). The ROC curve comparison of these three markers is shown in (Fig. 1).

Among 173 patients, there were 48 patients with MPVD of not less than 1.5 fL, 22 patients with stent restenosis within one year, and the rate of restenosis was 45.8%. In addition, there were 125 patients with an MPVD of < 1.5 fL, and 12 patients with stent restenosis within one year, and the rate of restenosis was 9.6% (*P* < 0.05). There were 49 patients with an MPVDR of not less than 17.9%, and 21 patients with restenosis that was evident within one year, and the rate of restenosis was 42.9%. There were also 124 patients with an MPVDR of < 17.9%, and 13 patients with stent restenosis seen within one year – the rate of restenosis was 10.5% (*P* < 0.05). The pre- and post-operative mean platelet volume difference of not less than 1.5 fL (*P* < 0.001), and the pre- and post-operative mean platelet volume difference ratio of not less than 17.9% percent (*P* < 0.001), were statistically different between the ISR and no-restenosis groups (Table 4).

**Fig. 2:**
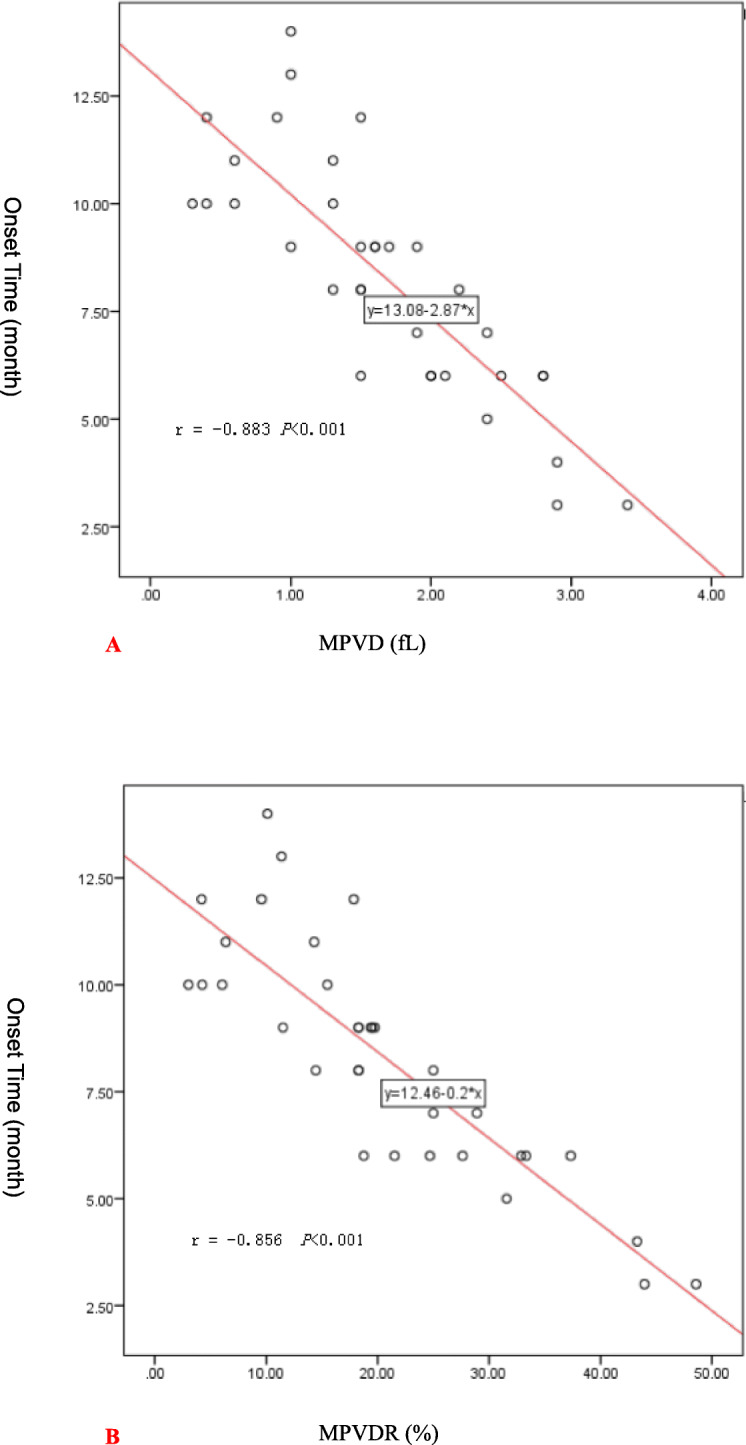
**a**: Pearson correlation analysis chart of MPVD and ISR onset time. **b**: Pearson correlation analysis chart of MPVDR and ISR onset time

There was a correlation between post-operative MPV and the occurrence of ISR (r = 0.58; *P* < 0.001; Table 5). A MPVD value of not less than 1.5 fl was associated with an odds ratio of 9.17 (95% CI [3.76 to 22.35]; *P* < 0.001) for the occurrence of ISR. A MPVDR value of not less than 17.9% was associated with an odds ratio of 7.68 (95% CI [3.19 to 18.49]; *P* < 0.001) for the occurrence of ISR (Table 6). Pearson’s correlation analysis showed that MPVD was negatively correlated with the onset time of ISR (r = − 0.856; *P* < 0.001). The greater the MPVD, the shorter the onset time of restenosis (Fig. 2a). MPVDR was also negatively correlated with the onset time of ISR (r = − 0.883; *P* < 0.001). The larger the MPVDR, the shorter the onset time of restenosis (Fig. 2b).

**Table 5:** Correlation coefficients and *p* values of Spearman rank regression analyses for the occurrence of ISR

Variables	r	*P*	Variables	r	*P*
Postoperative MPV	0.58	< 0.001	Preoperative lymphocyte ratio	0.16	0.035
Preoperative MPV	0.11	0.17	Preoperative neutrophil ratio	0.20	0.007
Preoperative platelet count	0.16	0.03	Preoperative neutrophil count	0.15	0.04
Postoperative platelet count	0.32	< 0.001			

Key: *MPV* mean platelet volume

**Table 6:** Multivariable analysis of predictors of ISR after superficial femoral artery stenting

Variables	MPVD		MPVDR	
	OR(95%CI)	*P* value	OR(95%CI)	*P* value
Neutrophil	1.24 (0.87–1.87)	0.224	1.22 (0.85–1.73)	0.269
Neutrophil ratio	1.06 (0.95–1.18)	0.258	1.07 (0.96–1.19)	0.195
Lymphocyte ratio	1.02 (0.91–1.15)	0.653	1.03 (0.92–1.15)	0.587
PCD	1.01 (1.00–1.02)	0.011	1.01 (1.0–1.02)	0.008
NLR	1.58 (1.32–1.81)	0.030	1.65 (1.44–1.98)	0.040
MPVD≥1.5 fL	9.17 (3.76–22.35)	< 0.001	–	–
MPVDR≥17.9%	–	–	7.68 (3.19–18.49)	< 0.001

Key: *PCD* platelet count difference, *NLR* neutrophil–lymphocyte ratio, *MPVD* mean platelet volume difference, *MPVDR* mean platelet volume difference ratio

**Fig. 3:**
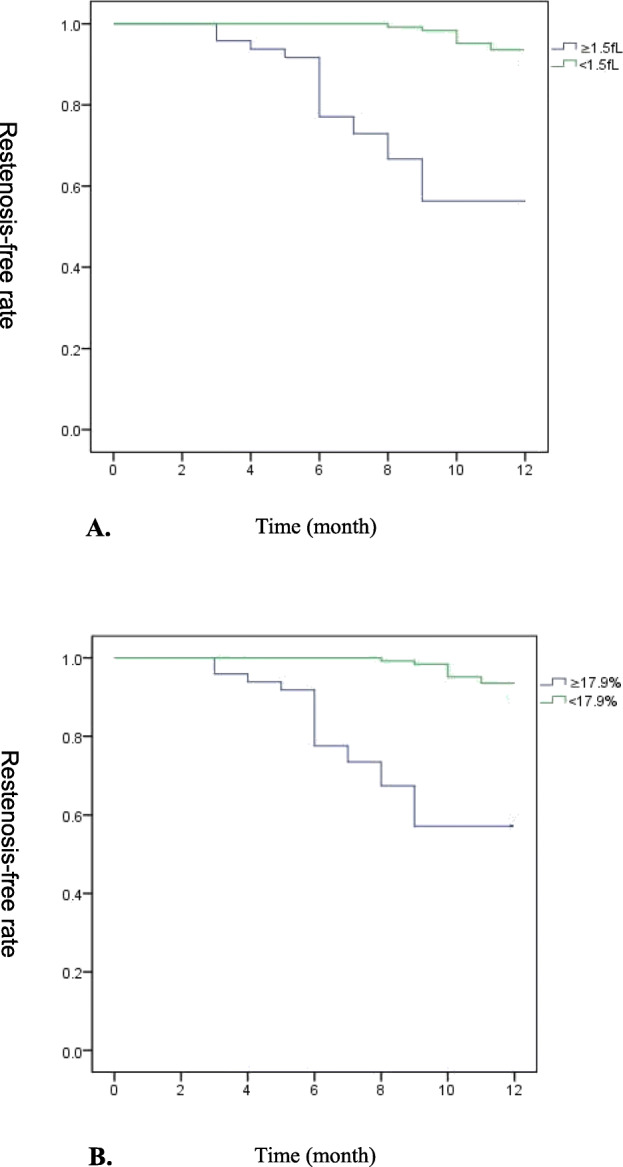
**a**. Cumulative freedom from in-stent restenosis with Kaplan–Meier analysis. Patients with an MPVD of less than1.5 fL are displayed by the green line; Patients with an MPVD not less than 1.5 fL are displayed by the blue line. Cumulative rates of freedom from in-stent restenosis were compared by the log-rank test (*P* < 0.001). **b**. Cumulative freedom from in-stent restenosis with Kaplan–Meier analysis. Patients with MPVDR less than 17.9% were displayed with green line; Patients with MPVDR not less than17.9% were displayed with blue line. Cumulative rates of freedom from in-stent restenosis were compared with log-rank test (*P* < 0.001)

The Kaplan–Meier analysis of cumulative freedom from ISR according to MPVD (≥1.5 fL or < 1.5 fL) is shown (Fig. 3a). The log-rank test indicated that ISR risk was significantly higher in patients with an MPV of not less than 1.5 fL than patients with an MPV of less than 1.5 fL at baseline (*P* < 0.001). Figure 3b shows the Kaplan–Meier analysis of cumulative freedom from ISR according to the MPVCR value (≥17.9% or < 17.9%). The log-rank test indicated that the risk of ISR was significantly higher in patients with MPVDR of not less than 17.9% than patients with MPVDR of less than 17.9% fL (*P* < 0.001).

## Discussion

In our study, the one year restenosis rate of the superficial femoral artery stent was 19.6%, which was consistent with other research findings.

Our study observed that in patients presenting with restenosis within 12 months, the MPV increased after stent implantation. Norgaz et al. [17] reported that a pre-operative MPV > 8.4 fL was associated with restenosis within six months after coronary stent implantation and there was a positive correlation between a preoperative MPV and the occurrence of ISR (r = 0.44; *P* < 0.001). However, the relationship between post-operative platelet volume and stent restenosis has not been analyzed. Dai et al. [18] reported that a pre-operative MPV of more than 10.1 fL was associated with restenosis within 16 months after carotid stent implantation (*P* = 0.013). There was no significant difference between the pre- and post-operative MPV in CAS patients.

We observed that the pre-operative MPV in the ISR group was 8.49 ± 0.91 fL compared to 8.31 ± 0.82 fL in the no-restenosis group (*P* > 0.05); however, the post-operative MPV in the ISR group was 10.04 ± 0.68 fl as compared to 9.11 ± 0.79 fl in the no-restenosis group (*P* < 0.001). There was no correlation between the pre-operative MPV and an occurrence of ISR (r = 0.11; *P* > 0.05). However, we did find a positive correlation between the post-operative MPV and an occurrence of ISR (r = 0.58; *P* < 0.001). Patients with an MPVD of not less than 1.5 fL had a 9.17-fold higher risk of ISR when compared with an MPVD of less than 1.5 fL. Moreover, patients with an MPVDR of not less than 17.9% had a 7.68-fold higher risk of ISR than did patients with an MPVDR of less than 17.9%. Further, Hu et al. [21] reported that patients with a higher platelet distribution width, defined as more than 13.65%, had a four-fold higher risk of ISR as compared with a lower platelet distribution width after coronary stent implantation.

Our study found no correlation between the platelet distribution width and an occurrence of ISR. These discrepancies might be caused by the different lesion sites studied. The superficial femoral artery stent might be affected by the compression, pulling and torsion of the thigh muscle, while the coronary and carotid arteries are less affected by the muscle. In addition, the blood flow velocity differs among that seen for the superficial femoral artery and the coronary and carotid arteries. We found that changes in the MPV for patients with restenosis have a negative linear relationship with the onset time of restenosis. It is suggested that the greater the change in the MPV during pre- and post-operative superficial femoral artery stent implantation, the shorter the onset time of restenosis. In addition, our study observed an increase in MPV and a decrease in platelet count in patients with restenosis.

Inoue et al. [22] reported 48 patients with left anterior descending coronary artery disease that were randomly assigned to either a balloon angioplasty group or a coronary stent group. The heparin-coated catheter remained in the coronary sinus for 48 h after the procedure. Coronary sinus blood and peripheral blood were obtained, before, immediately after, and at 24 and 48 h after the procedure. By flow cytometric analysis, it was demonstrated that in the coronary sinus of the stent group, the expression of platelet CD62P increased significantly, and did so immediately after the procedure, and these trends continued during the 48-h observation period. By contrast, in the coronary sinus of the balloon group, the expression of platelet CD62P increased less significantly when measured immediately after the procedure, and declined to baseline levels 24 h after the procedure. This result suggested that platelet activation that occurs in the coronary circulation after coronary stenting was both stronger and more persistent than following the balloon angioplasty procedure. Platelets do not have any nuclei, and their size and α-granule content are controlled by megakaryocyte development and differentiation [23].

In one particular animal study, Martin et al. [24] reported observations from a rabbit model following the intravenous daily injection of anti-rabbit platelet serum for six continuous days. At the end of the six day period, multiple fragments of bone marrow from the femoral shafts were taken. Megakaryocyte were measured using a Kontron MOP AM03 system. It was shown that the volume of megakaryocyte cytoplasm significantly increased and stimulated platelet production. The platelet volume produced per megakaryocyte also increased. Large platelets contain more α-granules. Moreover, platelet α-granules contain PDGF, which is thought to be the most potent mitogen in the process of smooth muscle cell hyperplasia, and the occurrence of neointimal hyperplasia [8].

Inoue et al. [25] measured serum levels of glycosyl-phosphatidil-inositol-anchored-protein-80, which is a modulator of Mac-1 on the surface of neutrophils in patients that had undergone coronary stent implantation. This group reported activation of neutrophils and neutrophil-mediated oxidative burst in the inflammatory process of PCI-induced vessel injury and neointimal hyperplasia. Chang et al. [26] recruited 180 patients and obtained venous blood samples one to three days before superficial femoral artery stent implantation. The neutrophil-lymphocyte ratio was computed using the absolute neutrophil count divided by the absolute lymphocyte count. This group observed that the neutrophil-lymphocyte ratio was significantly higher in the early ISR (i.e., within one year) group than that seen in the non-ISR group. The neutrophil-lymphocyte ratio that was not less than 3.62, was independently and positively associated with a higher risk of early ISR after stent implantation in patients with femoro-popliteal chronic total occlusion.

However, we found that the neutrophil-lymphocyte ratio was negatively correlated with ISR. This discrepancy is likely due to the patients in our study that did not have severe limb ischemia. However, Verdoia et al. [27] reported that in patients undergoing coronary angiography, the neutrophil-lymphocyte ratio is independently associated with ISR. In a study using coronary arteries of swine, Nakatani et al. [28] reported that neointimal hyperplasia after stenting lasts longer than is seen following balloon injury, presumably due to the inflammatory cells that are found around the stent struts. Inflammatory cells, such as macrophages and eosinophils, are intimately linked to the differences seen in neointimal hyperplasia following balloon injury as compared stenting. We found that post-operative cell counts of neutrophils, leukocytes, eosinophils, and basophils were all higher than was found preoperatively.

There are several limitations in the present study. First, this study was conducted on a retrospective basis, like other studies, represented a single center experience. Second, the limitations of our study was the small sample, which we recognize might lead to differences in some of the reported observations. Thied, the predictive effect of the serum inflammatory factor concentrations on restenosis after stent implantation was also neglected, which we recognize will need to be confirmed by further studies. In addition, although there are many factors (procedure related factor, underlying vascular disease factor, etc) which can affect in-stent restenosis (ISR), these factors were not fully evaluated in this study.

## Conclusion

In conclusion, the increase in pre- and post-operative stent implantation MPV was positively correlated with the occurrence of superficial femoral artery in-stent restenosis. When an MPVD of not less than 1.5 fL is found, or when an MPVDR of not less than 17.9% is found, the appearance of superficial femoral artery stent restenosis is more likely to occur.

## Data Availability

All data generated or analysed during this study are included in this published article.

## Abbreviations

MPV: Mean platelet volume
ISR: In-stent restenosis
SFAS: Superficial femoral artery stenting
MPVD: Mean platelet volume difference
MPVDR: Mean platelet volume difference ratio
PDGF: Platelet-derived growth factors
